# Long-term effect of radiofrequency turbinoplasty in nasal obstruction

**DOI:** 10.1080/13102818.2014.909083

**Published:** 2014-07-10

**Authors:** Mehmet Akdag, Suleyman Dasdag, Fazil Emre Ozkurt, Mehmet Yusuf Celik, Aydin Degirmenci, Huseyin Demir, Faruk Meric

**Affiliations:** ^a^ Department of Otolaryngology, Head & Neck Surgery, Medical School, Dicle University, Diyarbakir, Turkey; ^b^ Department of Biophysics, Medical School, Dicle University, Diyarbakir, Turkey; ^c^ Department of Biostatistics, Medical School, Dicle University, Diyarbakir, Turkey; ^d^ Akademi, Private Otolaryngology, Head & Neck Surgery, Diyarbakir, Turkey

**Keywords:** radiofrequency, inferior turbinate, nasal obstruction

## Abstract

The aim of this retrospective study is to investigate long term (two years) effect of radiofrequency tissue volume reduction (RFVTR) on nasal obstruction associated with inferior turbinate hypertrophy, which is not respond to medical treatment. This retrospective study carried out on 98 patients with nasal obstruction treated by RFVTR (56 males, 42 females, from 17 to 70 years of age). Visual analogue scales (VAS) and nasal endoscopic view score (NES) were used for nasal obstruction to evaluate the efficacy of the treatment. Results of one, three, six, twelve and twenty four months after RFVTR treatment were compared with pre-exposure values. Turbinate edema and nasal obstruction in the treated patients were recovered after one month of treatment (*p* < 0.01). Maximum improvement were determined at the end of third month (*p* < 0.01). However, eficacy of RFVTR declined twelve months after treatment. Final percentage of imrovement was found as 51 % at the end of second year of treatment because of co-factors was not eliminated by some patients. On the other hand, no statistical significant difference was observed between the right and left nasal cavity (*p *< 0.001). In conclusion, the result of this study indicated that patients who have not eliminate co-factors such as smoking, obesity and allergic rhinitis may need re-application of RFTVR. However, further studies on radiofrequecy energy level and elimination of other co-factors are necessary to illuminate the eficacy of RFVTR.

## Introduction

Nasal obstruction is a common problem among patients presenting to the general otolaryngologist. There are many potential causes for nasal obstruction. Some of the most common are turbinate hypertrophy, deviation of the nasal septum, nasal allergies, sinus or nasal infection, mass in the nasal cavity and idiopathic rhinitis. The vast majority of cases with nasal obstruction are due to turbinate hypertrophy.[1,2] Enlarged turbinates can impair normal breathing, causing patients to breathe through the mouth. Enlarged turbinates may be treated with intranasal sprays and medications. If turbinate hypertrophy is chronic, surgical intervention may be considered. Many different surgical techniques for the reduction of turbinates have been described, and their effectiveness has been compared, such as submucosal reduction (partial or total), cryosurgery, laser-assisted reduction, microdebrider-assisted submucosal reduction, outfracture, ultrasound reduction and radiofrequency tissue volume reduction (RFVTR).[2–7] Although there is lack of consensus about an optimal surgical technique [3,5,6] it is obvious that it should be effective, with fewer adverse side effects, and should preserve the functional properties of turbinates.

The radiofrequency (RF) that ranges from 300 to 3000 kHz has been defined as a medium frequency. This range represents one of the several conventions that are used when defining this part of the electromagnetic spectrum. Biological effects of radiofrequency radiation have been studied since the turn of past century.[8,9]

However, in developed countries there has been a remarkable growth in number of processes and devices that utilize or emit RF radiation.[9] Among them is one of the recent methods that is used for treatment of nasal obstruction – the radiofrequency turbinoplasty.

The goals of inferior turbinate surgery are straightforward. The surgeon aims to maximize volumetric reduction of the turbinate with improvement in nasal obstruction, but to maintain nasal function, and minimize complications.

RF provides a new surgical tool, designed to create a well-circumscribed submucosal scar that heals normally without the removal of tissue. The target site is stably formed with tissue-reduced volume approximately three weeks post-operatively. Since Li et al. [10] first reported the effect of RFVTR on turbinate hypertrophy, the safety and efficacy of this procedure were well demonstrated with respect to not only subjective improvement of symptoms but also objective changes in the nasal function.

The studies on RFVTR turbinate surgery mostly describe a short follow-up period.[3,11,7] However, research of long-term efficacy of RF turbinate surgery and comparison between the efficiency for the right and left nasal cavities is limited. Therefore, the purpose of this study was to evaluate the long-term efficacy of RFVTR turbinoplasty with respect to the obstructive symptoms, patient satisfaction and long-term alterations after a two-year follow-up period.

## Materials and methods

### Study subjects

The study was performed on 98 patients with nasal obstruction and associated bilateral inferior turbinate hypertrophy that was refractory to medication treatment (topical corticosteroids, decongestants, antihistamines) at least for three months.

Patients treated in Akademi Private ENT Surgery Centre between January 2008 and April 2012 were evaluated in this study. The study was based on history of diagnosis, clinical examination, nasal endoscopy, imaging and allergic testing. The study was approved by the Review Board of the Dicle University Medical Faculty Hospital (17.08.2012/669) and informed patient consent was obtained from all patients.

### Inclusion criteria

Patients with persistent nasal obstruction associated with hypertrophy of the inferior turbinates were included in the current study. These were patients diagnosed with allergic rhinitis, chronic rhinosinusitis and obstructive sleep apnoea disease associated with turbinate hypertrophy. Criterion for the selection of patients was lack of response to the application of topical decongestant agents.

### Exclusion criteria

Patients suffering from acute rhinosinusitis, diabetes mellitus, severe nasal deformities, coagulopathy disorders, severe systemic diseases, nasal tumours and history of nasal radiotherapy were excluded. Patients with septal or turbinate surgery were also excluded.

### Surgical procedure

All viable treatment options were discussed with each patient before undergoing the procedure. Surgical intervention was advised for all patients who failed to respond to conservative medical treatment. The intervention was performed in our department, on an outpatient basis, after a written informed consent was obtained. All patients underwent RFVTR under local anaesthesia. Ten minutes before the treatment, we introduced cotton soaked with a 2% topical pontocaine (tetracain) solution in each inferior meatus. Afterward, we used a 24-gauge needle to inject a solution of 2% topical pontocaine (tetracain) followed by injection of 2 ml of 1% lidocaine in the anterior and medial part of the inferior turbinate. We used the G3 Gyrus radiofrequency generator (Gyrus ENT, ACMI, Olympus Corporation) with target parameters set at: temperature of 75 °C; power of 15 W; and RF energy of 350 J with a total energy of 1050 J to each turbinate. The active portion of the needle was inserted longitudinally, from a 0° endoscopic view, into the submucosa of the anterior, middle and posterior parts of the inferior turbinate. Each patient was discharged without any limitation of normal daily activities. Following surgery, patients did not receive systemic or topical steroids. No nasal packing was administered. We advised the patients to use acetaminophen post-operatively if needed, but we did not prescribe any antibiotic treatment.

### Evaluation

Patients were asked not to use oral or topical steroids, antihistamines, or decongestants during the follow-up period. Patients who completed the examinations on the day of the procedure and at the 1st, 3rd, 6th, 12th and 24th month follow-up visit were only enrolled in this study. Patient surveys were conducted using: a standard 0 to 10 visual analogue scale (VAS), with 0 representing no symptoms and 10, the most severe symptoms (severity of obstruction, frequency of obstruction, discharge, hyposmia, stuffiness, headache, snoring, sneezing, itchy nose, crusting); a nasal endoscopic score (NES) equal or greater than 1 (1 = small turbinate with no contact with septum or nasal floor; 2 = mild hypertrophic turbinate with contact with septum; 3 = moderate hypertrophic turbinate with contact with septum and nasal floor; 4 = severe hypertrophic turbinate with contact with septum, nasal floor and superior compartment with complete nasal blockage).

All surgical candidates were given surveys prior to any surgical intervention. Patients were scored with VAS and NES at pretreatment after three months, six months, one year and two years. A physical and endoscopic examination was performed to evaluate the inferior turbinates and assess for bleeding, crusting, ulceration and reduction of turbinate volume. Preoperative and post-operative (after 3, 6, 12 and 24 months) mean values of each left and right nasal cavity were statistically compared.

### Data analysis

All statistical analyses were performed using the SPSS program (PASW statistics 17.0; SPSS, Inc., IBM Corporation, Chicago, USA). The significance of the differences between the outcomes prior to and after the intervention was assessed using Student's *t-*test for repeated measures, and *p* values less than 0.05 were considered statistically significant. All possible pairwise mean comparisons were found significantly different according to time and intercepts by using repeated ANOVA test of general linear model followed by *post hoc* test Bonferroni (*p* < 0.001).

## Results and discussion

Mild congestion and crusting was observed in the noses of the patients that underwent radiofrequency during the first week after the procedure. Symptoms of obstruction started to improve in the third week, but full recovery was observed after 21 to 45 days. However, major complications such as bleeding were not observed before and after the RF application. Drying and crusting in the nose were observed only in five patients and one spontaneous reduction in the sense of rhinorrhoea (neural denervation) was observed in a patient that completely healed after three months. No adverse reactions such as bleeding, infection, adhesions, synechiae, bone necrosis, atrophic rhinitis, or olfactory change were encountered for two years after surgery in all patients.

**Figure 1.**
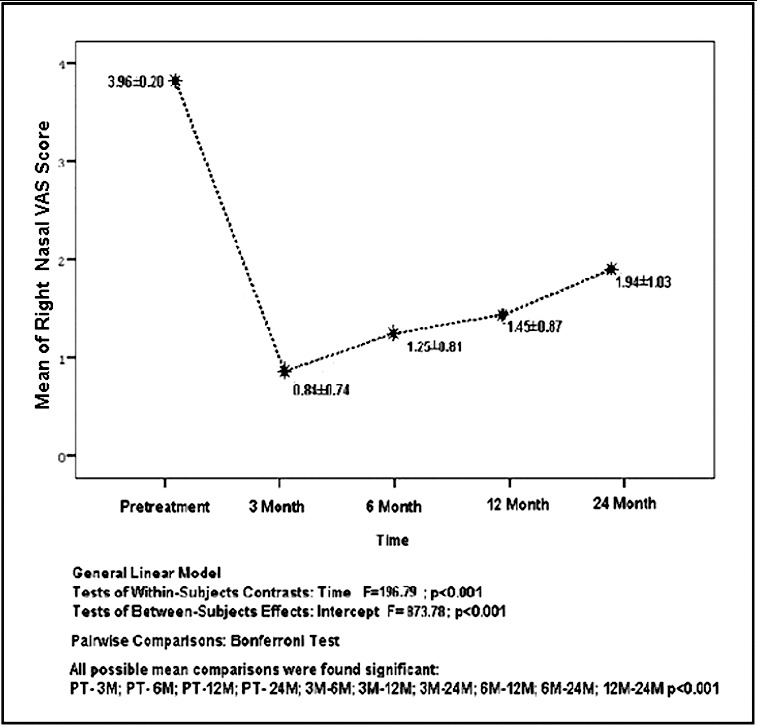
The distribution of the mean and standard deviation values of VAS for right nasal cavity according to time.

The mean and standard deviation values of VAS for the right nasal cavity at the following time points: pretreatment, 3rd, 6th, 12th and 24th months were, respectively, determined as 3.96 ± 0.20, 0.84 ± 0.74, 1.25 ± 0.81, 1.45 ± 0.87 and 1.94 ± 1.03, and are also shown in Figure 1. All possible pairwise mean comparisons were found significantly different according to time and intercepts by using repeated ANOVA test of general linear model followed by *post hoc* test Bonferroni (*p* < 0.001). The ratio of *improved nasal* obstruction was 79% from pretreatment to 3rd month, 68% from pretreatment to 6th month, 63% from pretreatment to 12th month and 51% from pretreatment to 24th month as the mean values were taken into consideration.

**Figure 2.**
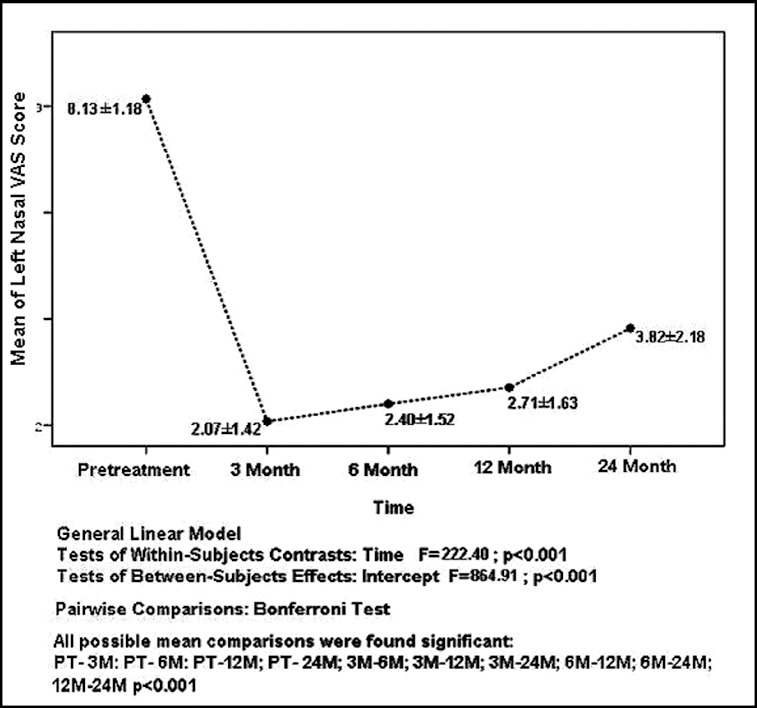
The distribution of the mean and standard deviation values of VAS for left nasal cavity according to time.

The mean and standard deviation values of VAS for the left nasal cavity at the following time points: pretreatment, 3rd, 6th, 12th and 24th months were, respectively, determined as 8.13 ± 1.18, 2.07 ± 1.42, 2.40 ± 1.52, 2.71 ± 1.63 and 3.82 ± 2.18, and are also shown in Figure 2. All possible pairwise mean comparisons were also significant (*p* < 0.001). The ratios of *improved nasal* obstruction were determined as 74% from pretreatment to 3rd month, 70% from pretreatment to 6th month, 66% from pretreatment to 12th month and 53% from pretreatment to 24th month as the mean values were taken into consideration.

**Figure 3.**
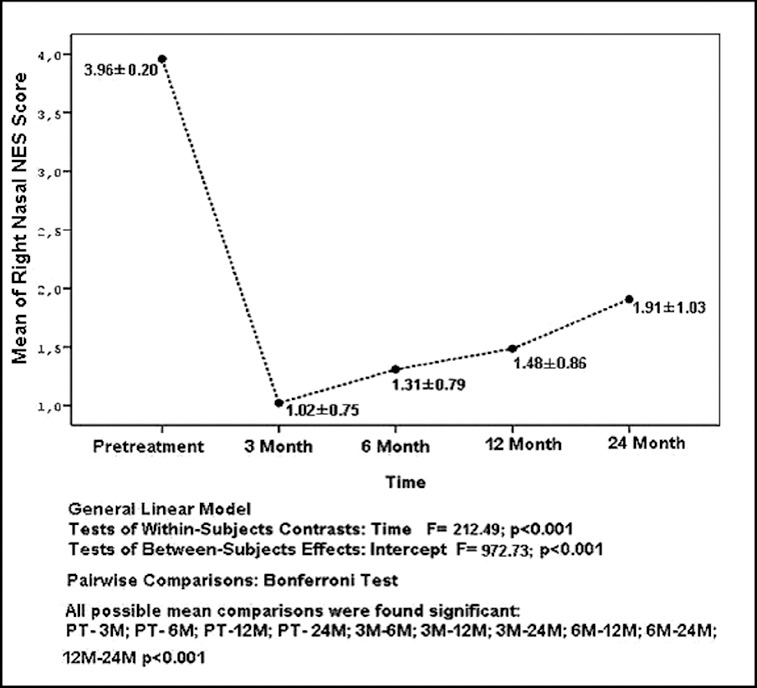
The distribution of the mean and standard deviation values of NES for right nasal cavity according to time.

The mean and standard deviation values of NES for the right nasal cavity were as follows: 3.96 ± 0.20, 1.02 ± 0.75, 1.31 ± 0.79, 1.48 ± 0.86, 1.91 ± 1.03 at pretreatment, 3rd, 6th, 12th and 24th months, respectively, and are also shown in Figure 3. The ratios for *improvement of the nasal* obstruction were established as 74% from pretreatment to 3rd month; 66% from pretreatment to 6th month; 62% from pretreatment to 12th month and 51% from pretreatment to 24th month by taking the mean values into consideration.

**Figure 4.**
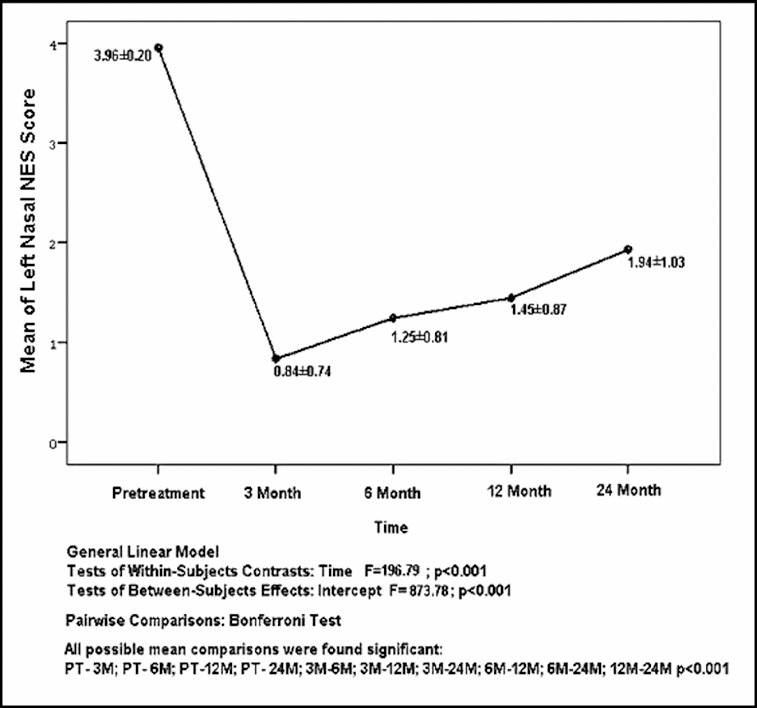
The distribution of the mean and standard deviation values of NES for left nasal cavity according to time.

The mean and standard deviation values of NES for the left nasal cavity were as follows: 3.96 ± 0.20, 0.84 ± 0.74, 1.25 ± 0.81, 1.45 ± 0.87, 1.94 ± 1.03 at pretreatment, 3rd, 6th, 12th and 24th months, respectively, and are also shown in Figure 4. The ratios for *improvement of the nasal* obstruction were established as 78% from pretreatment to 3rd month; 68% from pretreatment to 6th month; 63% from pretreatment to 12th month and 51% from pretreatment to 24th month by taking the mean values into consideration.

**Figure 5.**
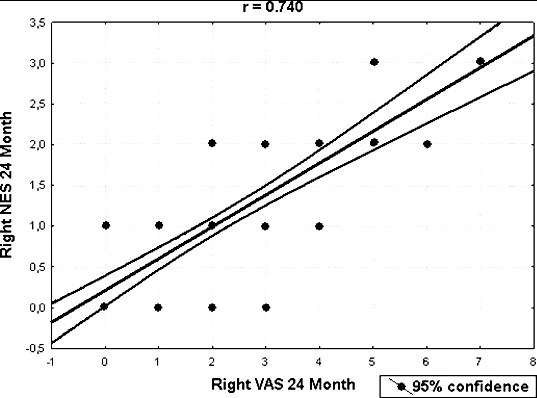
The distribution of NES and VAS for right nasal cavity after 24 months.

**Figure 6.**
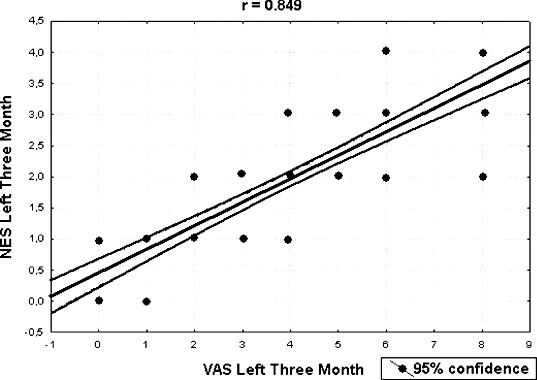
The distribution of VAS and NES for left nasal cavity after three months.

**Figure 7.**
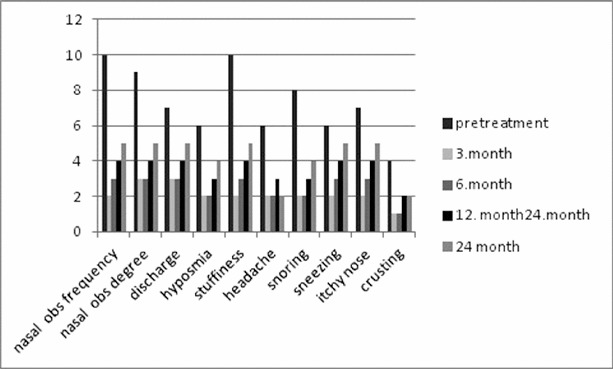
Subjective symptoms at preoperative and post-operative 3, 6, 12 and 24 months according to VAS.

All possible pairwise mean comparisons of data shown in Figures 3 and 4 were also significant (*p* < 0.001). The distribution of VAS and NES for the right nasal cavity after 24 months is shown in Figure 5. The correlation coefficient between NES and VAS for the right nasal cavity after 24 months was estimated as 0.740 and significant (*p* < 0.001). The distribution of NES and VAS for the left nasal cavity after three months was shown by Figure 6. The correlation coefficient between NES and VAS for left nasal cavity at the 3rd month was found as 0.849 and significant (*p* < 0.001). The ratios of improved symptoms were as follows: for nasal itching, 32%; headache, 68%; crusting, 50%; hyposmia, 32%; snoring, 51%; and sneezing, 56%, from pretreatment to the 24th month, respectively. The subjective nasal symptoms at preoperative and post-operative 3, 6, 12 and 24 months according to VAS are shown in Figure 7.

RF thermal energy induces an ionic agitation with vapourization at the cellular level in tissues. These processes lead to fibrotic changes and reduction of volume in tissues during the healing period.[12] RF surgery is usually well accepted by patients because of low rate of associated complications and high efficiency of the treatment. However, the most important points are permanence and sustainability of the treatment results. In a prospective, randomized clinical trial, Sapci et al. [13] compared nasal functions of patients with chronic nasal obstruction following treatment by: (1) radiofrequency tissue ablation; (2) laser ablation; and (3) partial turbinectomy. They reported that RFVTR was more effective for improvement of the nasal obstruction and in preservation of nasal mucociliary function. Laser ablation of the turbinate was also found to be very effective in terms of improving the nasal obstruction, whereas it obviously disturbed the mucociliary function. Partial turbinectomy resulted in a similar rate of improvement as that of radiofrequency tissue ablation. However, these findings were based on a small sample size and a relatively short follow-up (12 weeks).

Two different aspects of our study are related to: first, the clinical effectiveness of RTVR that has been evaluated in a long follow-up (two years) and second, it compares the results between the right and left nasal cavity.

The analysis of the results showed that RFVTR for inferior turbinate hypertrophy was effective during the two-year follow-up period. The percentage of efficacy for RFVTR without any complications was found to be 76% at the end of the third month after the treatment. Some improvement of obstructive symptoms was also observed in the remaining 24% of the treated patients at the same period. However, the efficacy of RFVTR decreased to 51% at the end of the second year. We believe that the decrease was attributed to various co-factors (multifactorial) such as smoking, reflux, obesity, allergic rhinitis, etc. that were seen in the relapsed patient. On the other hand, we believe that another reason for the relapse is the frequency of treatment session as only one procedure was applied during the two-year period. It may be suggested that RFVTR procedures are repeated quarterly during the treatment period. Moreover, the relapse of the patients’ symptoms may have originated from the applied amount of RF energy. In total 1050 J has been applied to the conchae of the patients with hypertrophic turbinates. In comparison, Cukurova et al. [14] applied 450–480 J for each turbinate and indicated that the RF procedure that was used by them was effective for inferior turbinate hypertrophy (82% during five years). However, the researchers did not provide information concerning the elimination of co-factors. Although different RF energy levels were applied, the results from their study were in support to our data when VAS values were compared. The results of Cukurova et al. support our hypothesis that increasing the RF energy levels and the frequency of RFVTR sessions may be beneficial and should be an issue for future research. Matthew et al. [15] reported that RFVTR is very effective for improvement of nasal obstructions in a long-term follow-up of two years. However, the weakness of their study was the size of the study group which was limited to 19 patients. In contrast, the number of patients included in the current study was 98. Also, Matthew et al. [15] did not report the number of treatment sessions, the amount of applied RF energy and the presence of co-factors. It is also shown by us that RFVTR was a very effective tool for the treatment of patients that did not have any co-factor. Thus, good compliance and cooperation with the patients with nasal obstruction is another important factor in the complex treatment. For instance, a very good cooperation was established between us and some of the patients with obesity and that used to smoke. These patients were convinced to quit smoking and lose weight during the two-year period and a relapse in their symptoms was not observed.

Strong correlation between nasal congestion score of patients and physical or anatomical findings was observed by us and was another important result supported by the obtained VAS and NES. A statistically significant relationship between VAS and NES data during the two-year period (Figures 5 and 6) was established. The most widely used tool for evaluation of nasal obstruction is the VAS. It has been shown that the VAS is sensitive to small changes in the health status after therapy. It also has been indicated that VAS is a single-item scale, where the type of the used scale and the construction of the question associated with the scale can dramatically influence the precision of the cross-sectional measurement of a symptom.[16,17] These tests have not been demonstrated to be superior compared to physical examination, nasal endoscopy or CT imaging, for selection of patients who would benefit from medical and/or surgical management of their nasal obstruction. The roles of AR and RMM still have to be proven.[18] The perception of nasal airflow is primarily a subjective sensation. However, since many factors may be of influence, no single objective test, although qualitatively and technically reliable, may reproducibly correlate with this perception. Andre et al. [19] reported in a systematic review that instruments mostly used for assessment of the subjective sense of nasal obstruction were not reliable and the used questionnaires were not validated. Moreover, opinions concerning the value of objective measurements of nasal patency in the clinical practice continue to disagree. Zhang et al. [20] reported that clinician assessment of nasal airflow had significant positive correlation with patients’ VAS, and the two methods significantly correlated with the parameters of rhinomanometry and acoustic rhinometry. Matthew et al. [15] reported long-term results after radiofrequency treatment of inferior turbinate hypertrophy by only using VAS analysis. While VAS can measure the patient's subjective complaints, NES is an important diagnostic tool that allows the physician to directly visually evaluate the obstruction status. Both VAS and NES methods were used by us when comparisons between the patients were made. These two methods are commonly used for evaluation of hypertrophic inferior turbinates.[11,15,21] Kjaergaard et al. [22] also reported an association between VAS and physiological nasal parameters measured by peak nasal inspiratory flow and acoustic rhinometry, which was another approach to the issue. As we could not use rhinomanometry it is considered a limitation of the current study.

Luczaj et al. [11] showed that RFVTR was equally efficient for both the right and left nasal cavities. Similarly, our results were in agreement. However, it was stated that bipolar radiofrequency thermal ablation was an effective method for the therapy of turbinate hypertrophy.

## Conclusions

RFVTR is a very effective and simple method for the treatment of patients with nasal obstruction. It allows patients to be managed in office environment. We believe that increasing the number of treatment sessions and the amount of RF energy, together with elimination of co-factors will improve the effectiveness of the nasal obstruction therapy. However, additional studies in order to verify our hypothesis are needed.
